# Predictors of Response for “Off” Time Improvement With Levodopa-Carbidopa Intestinal Gel Treatment: An Analysis of the GLORIA Registry

**DOI:** 10.3389/fneur.2020.00419

**Published:** 2020-06-19

**Authors:** Werner Poewe, Lars Bergmann, Weining Z. Robieson, Angelo Antonini

**Affiliations:** ^1^Medical University of Innsbruck, Innsbruck, Austria; ^2^AbbVie Inc., North Chicago, IL, United States; ^3^Department of Neuroscience, University of Padua, Padua, Italy

**Keywords:** LCIG, Parkinson's disease, levodopa, predictors, response, routine care, registry

## Abstract

**Background:** Levodopa-carbidopa intestinal gel (LCIG) is a long-term therapy for motor fluctuations in patients with advanced Parkinson's disease (PD). The aim of this analysis was to identify the baseline characteristics that predict “Off” time reduction in advanced PD patients treated with LCIG under routine clinical care in the GLORIA registry.

**Methods:** Patients were followed under routine care for 24 months (M) with delivery of LCIG via percutaneous gastrojejunostomy. Analysis of covariance (ANCOVA) and logistic regression were performed to identify baseline characteristics that predict “Off” time reduction.

**Results:** Compared to baseline, 86% (n/N = 131/152; mean ± SD baseline “Off” time: 3.4 ± 2.2 h) of M24 completers had ≥ 1 h reduction in “Off” time and 64% (n/N = 97/152; mean ± SD baseline “Off” time: 7.6 ± 2.9 h) had ≥ 3 h “Off” time reduction at M24. Most baseline characteristics were similar across responder subgroups; however, patients with ≥ 3 h “Off” time improvement had more “Off” time and less time with dyskinesia at baseline compared to patients with <3 h “Off” time reduction. Despite having less improvement in absolute “Off” h at M24, patients with <3 h “Off” time reduction experienced a 33% median reduction in “Off” time and a 44% median reduction in dyskinesia duration at M24, which was similar to the dyskinesia improvement observed among patients with ≥ 3 h “Off” time improvement (50% median reduction). Baseline “Off” time was both the best predictor of and the only significant factor associated with “Off” time improvement (*P* <0.0001).

**Conclusions:** LCIG treatment led to clinically meaningful improvements in “Off” time in 86% of advanced PD patients and those with greater “Off” time are likely to experience the largest absolute reduction in hours “Off.”

## Introduction

Early stages of Parkinson's disease (PD) are well-controlled with standard oral levodopa therapy; however, long-term oral treatment is associated with the development of disabling motor and non-motor fluctuations. In patients with advanced PD, providing an optimal dose of levodopa that controls “Off” time without inducing disabling dyskinesia and/or non-motor fluctuations is challenging and, in many patients, eventually requires advanced therapeutic options (1–5). One long-term treatment option for advanced PD patients is levodopa-carbidopa intestinal gel (LCIG, carbidopa-levodopa enteral suspension in the United States [CLES]) that is continuously delivered to the upper intestine via percutaneous gastrojejunostomy (PEG-J) and a portable infusion pump. Previous studies have demonstrated the efficacy of LCIG infusions for reducing the motor fluctuations and non-motor symptoms that many levodopa-treated advanced PD patients experience (6–9). One critical data gap is determining when to transition a patient to a more advanced therapy and baseline characteristics that would predict a favorable response to LCIG therapy.

To date, little information is available regarding the patient characteristics that best predict the magnitude of response to LCIG therapy. A recent publication that evaluated LCIG-treated patients from a phase 3 clinical trial reported that a patient's baseline “Off” time was a strong predictor of whether or not a patient experienced clinically meaning improvements in “Off” time over the course of treatment (10). These analyses were performed in clinical trials and it is unknown if this observation also applies to the use of LCIG in routine clinical practice. Providing clinicians and patients with real world data (11) and predictors of response would help guide the transition to advanced therapeutic options, such as LCIG.

The “Off” time responder analysis reported here evaluated the baseline characteristics that are predictive of ≥ 3 h “Off” time reduction in advanced PD patients treated with LCIG under routine clinical care using data from the observational GLORIA registry (9). Unlike controlled clinical trials, the GLORIA registry was a real world registry without restrictive inclusion/exclusion criteria beyond those required by local labeling and national reimbursement criteria. Additionally, the GLORIA registry was conducted in nearly all of the countries where LCIG was available (registry conducted 2010–2015) and was designed to replicate the monitoring frequency of routine clinical care, with follow-up visits conducted every 6 months. In this analysis, “Minimal clinically meaningful improvement” was defined as having ≥ 1 h improvement in “Off” time compared to baseline at treatment month 24 and was derived from the minimal clinically important change determined by Hauser et al. (12). “Robust” responders were defined as patients having ≥ 3 h improvement in “Off” time compared to baseline at month 24 and was selected as an improvement threshold that reflects a defined substantial clinical difference in advanced PD patients (10, 13, 14).

## Materials and Methods

### Study Design and Patients

The current report is a *post hoc* analysis of the GLORIA registry (Global LOng-term Registry on efficacy and safety of LCIG in patients with Advanced Parkinson's disease in routine care), which was a 24-month, non-interventional, observational registry that enrolled male and female advanced PD patients with persistent motor complications at 75 movement disorder centers across 18 countries. National and/or local independent ethics committees at each participating institution approved the protocol. All patients provided written informed consent. LCIG treatment was initiated via an optional temporary nasojejunal (NJ) tube for dose optimization and then administered through PEG-J (according to European Commission Summary Product Characteristics and national reimbursement criteria). Clinical observations were recorded prospectively for up to 24 months for LCIG-naïve patients. For patients who had received LCIG for ≤ 12 months before enrollment in the registry, clinical observations were collected retrospectively up to the day of registry enrollment and then prospectively for a total observation period of 24 months. Complete registry design information and patient details, including the safety and tolerability results, are reported in Antonini et al. (9).

### *Post hoc* Analyses

Baseline efficacy assessments were collected before any LCIG-related procedure was performed and included the Unified Parkinson's Disease Rating Scale (UPDRS) parts II and III, the “Off” time and the dyskinesia items from UPDRS part IV, the Non-Motor Symptom Scale (NMSS), and the 8-item Parkinson's Disease Questionnaire (PDQ-8). UPDRS IV items 39 and 32 were modified by using the rating instructions for the corresponding parts 4.3 and 4.1 of the Movement Disorder Society (MDS)-UPDRS to collect the actual hours of “Off” time and “On” time with dyskinesias. Baseline daily levodopa equivalent dose (LED) was also evaluated and calculated using published conversion factors for the administration of LCIG and concomitant oral PD treatment at each study visit (15).

Only patients with baseline and month 24 efficacy assessments were included in this *post hoc* analysis (*N* = 152/375 enrolled patients). The change in total daily hours of “Off” time from baseline to treatment month 24 was used to define “Off” time responder subgroups: patients with “Off” time improvement from baseline to month 24 of <1 h and patients with an improvement from baseline to last visit of ≥ 1 h—the latter corresponding to the minimal clinically important change in “Off” time as defined by Hauser et al. (12). In addition, a subset of the responder subgroup of patients was analyzed that demonstrated a decrease in “Off” time of ≥ 3 h (defined as “robust” responders) from baseline to month 24 (10). Baseline demographics, clinical characteristics, and treatment effects were examined for the subgroups of patients with <3 and ≥ 3 h “Off” time reduction at month 24. An ANCOVA was performed to identify the baseline characteristics that are associated with treatment-related “Off” time improvements on patients with no missing baseline values (*N* = 118). A logistic regression analysis with backward selection of baseline characteristics was performed on “robust” responder status at month 24 (*N* = 118). The following demographic and baseline characteristics were included in both the ANCOVA and logistic regression model: age, PD duration, “Off” time, dyskinesia time, NMSS total score, and PDQ-8 summary index. Baseline characteristic parameters were removed from the model until all remaining parameters had a *p*-value of <0.2. For all remaining parameters in the model a *p*-value of <0.05 was set for significance.

## Results

**Figure 1:**
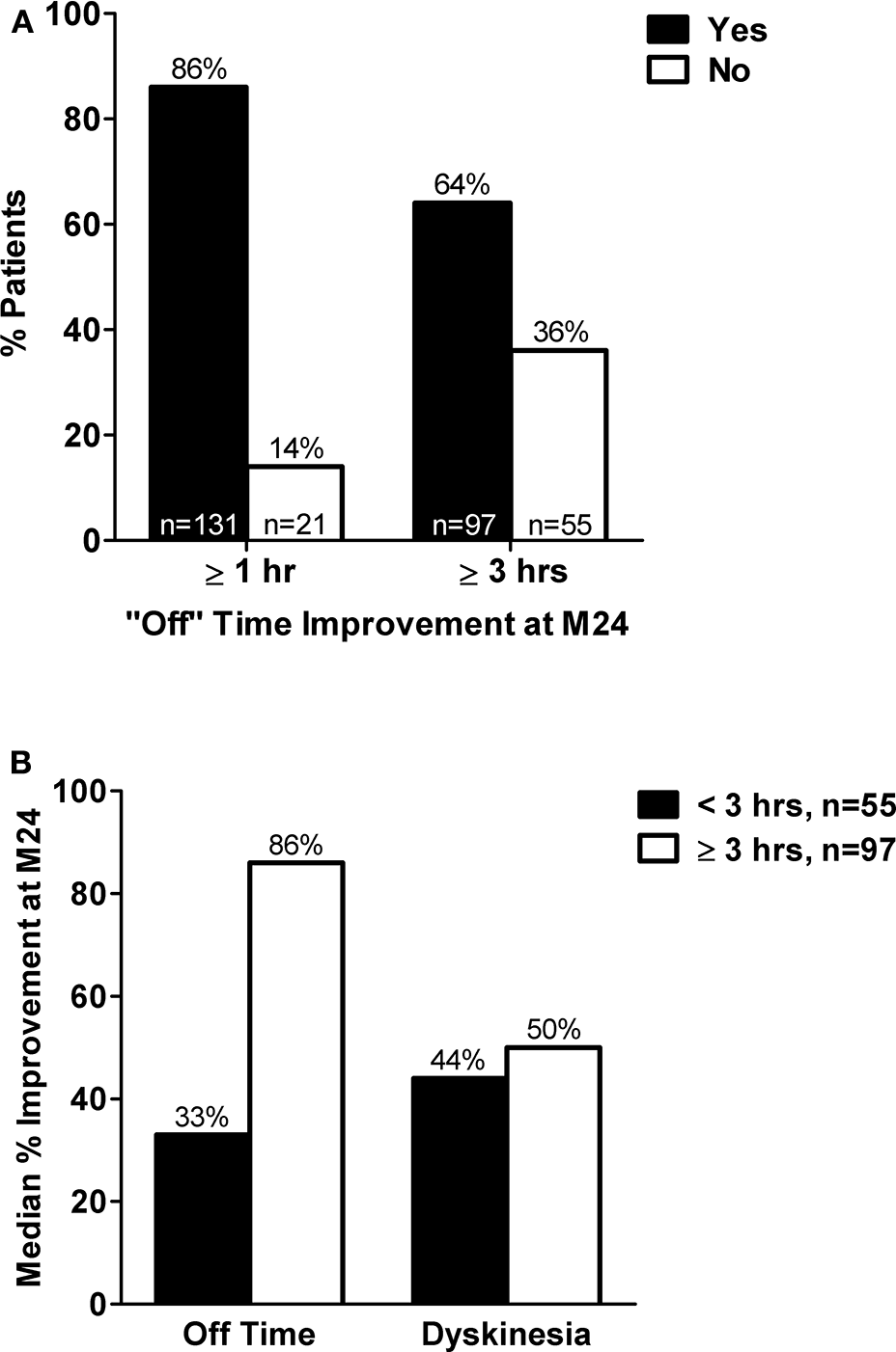
**(A)** Percent of patients with <1 h vs. ≥ 1 h and <3 vs. ≥ 3 h reduction in “Off” time at treatment month 24 compared to baseline. **(B)** Median percent improvement in hours of “Off” time and hours of dyskinesia at treatment month 24 compared to baseline in subgroups of patients with <3 or ≥ 3 h “Off” time improvement at month 24. M, month; hr(s), hour(s). Adapted from Poewe et al. (16)

A total of 375 patients were enrolled in the registry. Of the 152 patients included in this LCIG responder analysis, 86% (*n* = 131) met the criterion for minimal clinically meaningful “Off” time improvement (≥ 1 h reduction in “Off” time) at month 24 compared to baseline (Figure 1A). A majority (64%, *n* = 97) of the patients were “robust” responders and had a ≥ 3 h reduction in “Off” time at month 24 compared to baseline (Figure 1A). Patients with <3 h of “Off” time improvement from baseline at month 24 had less baseline “Off” hours and more “On” time with dyskinesia, as well as lower baseline NMS burden compared to patients (“robust” responders) with “Off” time improvement of ≥ 3 h (Table 1). Apart from these parameters the baseline characteristics were similar between the responder groups. Patients with <3 h “Off” time reduction had a 33% median reduction in “Off” time and a 44% median reduction in dyskinesia duration after 24 months of LCIG treatment (Figure 1B). “Robust” responders experienced a similar median reduction in dyskinesia duration (50% median reduction in dyskinesia at month 24) but their median reduction from baseline in “Off” time at month 24 was 86%. In accordance with the reduction in dyskinesia duration, compared to baseline at month 24 dyskinesia-related disability (UPDRS item 33 scores) improved in patients with <3 h of “Off” time improvement from 1.71 ± 1.08 to 0.82 ± 0.87 and decreased from 1.66 ± 1.22 to 0.82 ± 0.89 in the “robust” responders. Additionally, dyskinesia-related pain (UPDRS item 34 scores) decreased in the <3 h responders from 0.90 ± 1.05 to 0.25 ± 0.58 and decreased from 0.94 ± 1.08 to 0.26 ± 0.67 in the ≥ 3 h responders.

**Table 1:** Baseline characteristics among patients with <3 or ≥ 3 h “Off” time improvement after 24 months of LCIG treatment.

	**“Off” time improvement at month 24**	
	** <3 h^a^ (*n* = 55)**	**≥ 3 h (*n* = 97)**
**Baseline demographics and disease characteristics, mean (SD) [median]**		
Age, years	66.6 (7.8) [67.0]	64.7 (7.9) [66.0]
PD duration, years	12.4 (5.8) [11.8]	13.8 (7.0) [12.6]
LED, mg/day	1356.9 (822.1) [1225.0]	1356.9 (538.1) [1275.0]
Modified UPDRS IV item 32, dyskinesia hours	5.2 (4.0) [4.5]	3.9 (3.4) [4.0]
Modified UPDRS IV item 39, “Off” time hours	3.4 (2.2) [3.0]	7.6 (2.9) [7.0]
PDQ-8 summary index	43.9 (19.1) [46.9]	49.5 (19.2) [46.9]
NMSS total score	52.2 (36.3) [41.0]	71.1 (42.7) [71.0]
**Disease characteristics at month 24 of LCIG treatment, mean (SD)**		
**[median]**		
LED, mg/day	1700.1 (704.4) [704.4]	2045.5 (876.3) [876.3]
Modified UPDRS IV item 32, dyskinesia hours	2.7 (2.5) [2.5]	3.4 (4.0) [2.0]
Modified UPDRS IV item 39, “Off” time hours	2.7 (2.4) [2.0]	1.6 (1.7) [1.0]
PDQ-8 summary index	36.7 (19.5) [34.9]	41.5 (18.6) [37.5]
NMSS total score	49.8 (44.9) [36.0]	52.6 (37.3) [50.0]

*LED, levodopa equivalent dose; NMSS, non-motor symptom scale; PD, Parkinson's disease; PDQ-8, 8-item Parkinson's disease questionnaire; SD, standard deviation; UPDRS, unified Parkinson's disease rating scale*.

a * <3 h improvement subgroups include patients who worsened and patients with “Off” time improvement of <3 h. Adapted from Poewe et al. (16)*.

Baseline “Off” time was the only baseline characteristic that significantly correlated with treatment-related “Off” time improvement at month 24 in the ANCOVA (*N* = 118, *f* = 211.71, *P* < 0.0001). A logistic regression model with backward selection was applied to determine the predictive influence of various baseline characteristics for “robust” responders (≥ 3 h “Off” time improvement at month 24) with LCIG treatment. The logistic regression analysis again showed that baseline “Off” time was the strongest predictor of “Off” time improvement in “robust” responders (Table 2). In the model, baseline disease duration (longer), LED (lower), and PDQ-8 summary index (worse QoL) were other factors identified that related to “Off” time response but did not reach statistical significance (Table 2). The safety and tolerability results from the GLORIA registry were consistent with the known safety profile of LCIG (9).

**Table 2:** Results of the logistic regression model with backwards selection for “robust” responder status at month 24.

**Baseline characteristic**	**Estimate**	**Std. error**	**Wald chi-square**	***P*-value**
Disease duration	0.14	0.06	5.13	0.0235
Baseline LED	−0.00	0.00	2.80	0.0942
Baseline “Off” time hours (Modified UPDRS IV item 39)	1.4	0.29	23.95	<0.0001
Baseline PDQ-8 summary index	−0.03	0.02	1.88	0.1698

“*Robust” responder was defined as patients with ≥ 3 h of “Off” time improvement at month 24. Parameters were removed from the model if they reached a 0.2 significance level. LED, levodopa equivalent dose; PDQ-8, 8-item Parkinson's disease questionnaire; Std, standard; UPDRS, unified Parkinson's disease rating scale. Adapted from Poewe et al. (16)*.

## Discussion

The study presented here assessed baseline characteristics that might be predictive of response (reduction in “Off” time) to LCIG treatment in patients with advanced PD. The GLORIA registry, unlike previous analyses in controlled trials, allowed for these assessments in a substantial number of patients treated with LCIG during routine clinical care. The primary results from the GLORIA registry demonstrated LCIG to provide sustained reductions in “Off” time compared to baseline and tolerability that was consistent with the overall known safety profile of LCIG (9).

This *post hoc* analysis of the GLORIA registry support the high rate of LCIG treatment response reported for other studies (10, 17), with 86% of patients achieving a clinically meaningful reduction in “Off” time from baseline of ≥ 1 h after 24 months of LCIG treatment. Sixty-four percent of patients had “Off” time reductions of ≥ 3 h. Importantly, the improvement threshold of ≥ 3 h “Off” time improvement from baseline for “robust” responders exceeds the threshold for a minimally important clinical difference (≥ 1 h reduction in “Off” time) for advanced PD patients (12, 14). Under both improvement criteria, a majority of patients in either subgroup (≥ 1 h or ≥ 3 h in “Off” time reduction) were responders. These data are consistent with the phase 3 clinical trial responder rates reported by Standaert et al. (10); however, these data are derived from patients undergoing 2-years of open-label LCIG treatment in a routine clinical care setting, providing valuable data on responder rates in a real-world setting.

Baseline characteristics between subgroups were similar; however, a lower baseline burden of “Off” time and more baseline “On” time with dyskinesia was observed in patients who had <3 h of “Off” time improvement after treatment at month 24 compared to patients with ≥ 3 h “Off” time improvement (“robust” responders). However, despite these differences (lower baseline “Off” time and higher baseline dyskinesia), as a group, patients with <3 h “Off” time improvement still showed a 33% median improvement in “Off” time and a 44% median improvement in dyskinesia duration after 24 months of treatment, indicating a benefit with LCIG treatment despite lower absolute hours of “Off” time reduction. Both groups also experienced improvements in dyskinesia-related disability and pain independent of “Off” time reduction with LCIG. “Robust” responders also had higher baseline NMSS scores compared to patients with <3 h “Off” time improvement; therefore, these patients who spent more time in the “Off” state at baseline, likely reflects the NMS component of levodopa response fluctuations among advanced PD patients (18).

“Robust” “Off” time responders were observed across a range of baseline characteristics, but with the ANCOVA analysis a significant relationship was only observed between a patient's baseline “Off” time burden and their “Off” time response to LCIG treatment (“Off” time improvement). The logistic regression further confirmed the influence of baseline “Off” time in predicting a patient's motor response to LCIG treatment. Limitations of the study include the *post-hoc* nature of the analysis as well as the inherent design issues common to observational registries which in part resulted in a large number of enrolled patients did not have month 24 data. Even with these limitations, this responder analysis supports the only published responder analysis of LCIG treated patients to date (10) and extends those findings to include observations using real-world clinical evidence.

Overall, these data provide important real-world clinical evidence to confirm the efficacy of LCIG therapy for “Off” time reduction. These results are encouraging and show that patients with severe motor fluctuations in terms of amount of daily “Off” time are likely to show the most impressive response to LCIG therapy. Alongside previously published results from a phase 3 clinical trial (10), these data show that LCIG “Off” time responders are observed across a range of baseline characteristics, with only baseline “Off” time having a significant influence on “Off” time response to LCIG therapy. Furthermore, the rates of “Off” time response to LCIG treatment are high in this advanced PD patient population even when using the more higher response threshold of ≥ 3 h reduction in “Off” time from baseline. These data provide important clinical information related to LCIG patient selection and therapeutic response when considering advanced therapies.

## Data Availability Statement

AbbVie is committed to responsible data sharing regarding the clinical trials we sponsor. This includes access to anonymized, individual and trial-level data (analysis data sets), as well as other information (e.g., Protocols and Clinical Study Reports), as long as the trials are not part of an ongoing or planned regulatory submission. This includes requests for clinical trial data for unlicensed products and indications.

This clinical trial data can be requested by any qualified researchers who engage in rigorous, independent scientific research, and will be provided following review and approval of a research proposal and Statistical Analysis Plan (SAP) and execution of a Data Sharing Agreement (DSA). Data requests can be submitted at any time and the data will be accessible for 12 months, with possible extensions considered. For more information on the process, or to submit a request, visit the following link: https://www.abbvie.com/our-science/clinical-trials/clinical-trials-data-and-information-sharing/data-and-information-sharing-with-qualified-researchers.html.

## Ethics Statement

The studies involving human participants were reviewed and approved by National and/or Local Independent Ethics Committees at each participating institution approved the protocol. The patients/participants provided their written informed consent to participate in this study.

## Author Contributions

WP, WR, LB, and AA contributed to the concept and analysis of the data presented in the manuscript. WR performed the statistical analysis. All authors interpreted and discussed the results and contributed to and approved the final version of the manuscript.

## Conflict of Interest

WP was a study investigator and has received compensation from AbbVie, Astra Zeneca, Teva, Novartis, BIAL, Biogen, Britannia, NeuroDerm, UCB, Orion Pharma, Zambon, and Merz Pharmaceuticals (consultancy and lecture fees in relation to clinical drug development programs for PD) outside the submitted work. He has also received royalties from Thieme, Wiley-Blackwell, and Oxford University Press. LB and WR are employees of AbbVie and receive stock or stock options. AA was a study investigator and has received compensation for consultancy and speaker-related activities from UCB, Lundbeck, Bial, NeuroDerm, Boehringer Ingelheim, AbbVie, Mundipharma, and Zambon.
